# Supplementary material for: Antifungal Activity of a Library of Aminothioxanthones
Source: Antibiotics (Basel). 2022 Oct 27;11(11):1488. doi: 10.3390/antibiotics11111488 (PMC9686595; doi:10.3390/antibiotics11111488)
Supplement: Supplementary file 1 [file antibiotics-11-01488-s001.zip › antibiotics-1940731-supplementary.pdf]

Supplementary Information

Antifungal Activity of a Library of Aminothioxanthones

Joana Cardoso [1,2,3], Joana Freitas-Silva [2,3], Fernando Durães [1,3], Diogo Teixeira Carvalho [1,4], Luís Gales [5,6], Madalena Pinto [1,3,], Emília Sousa [1,3,*], and Eugénia Pinto [2,3,*]

[1] Laboratory de Organic and Pharmaceutical Chemistry, Department of Chemical Sciences, Faculty of Pharmacy, University of Porto, 4050-313 Porto, Portugal
[2] Laboratory of Microbiology, Department of Biological Sciences, Faculty of Pharmacy, University of Porto, 4050-313 Porto, Portugal
[3] Interdisciplinary Centre of Marine and Environmental Research (CIIMAR), University of Porto, 4450-208 Matosinhos, Portugal
[4] Laboratory of Research in Pharmaceutical Chemistry, Department of Food and Drugs, Faculty of Pharmaceutical Sciences, Federal University of Alfenas, 37137-001 Alfenas, Brazil
[5] Institute of Biomedical Sciences Abel Salazar (ICBAS), University of Porto, 4050-313 Porto, Portugal
[6] Institute of Molecular and Cellular Biology (i3S-IBMC), University of Porto, 4200-135 Porto, Portugal
* Correspondence: esousa@ff.up.pt (E.S.); epinto@ff.up.pt (E.P.)

Table of Contents:

S.1 - NMR spectra for compounds **8**, **9**, and **10**.

S.2 – HRMS spectra for compounds **8**, **9**, and **10**.

S.3 - X-ray analysis of intermediate **16**.

S.4 - Docking results for compound **1** and reference compounds into the enzyme-binding site of lanosterol 14-α-demethylase.

S.1 - NMR Spectra for compounds **8**, **9**, and **10**

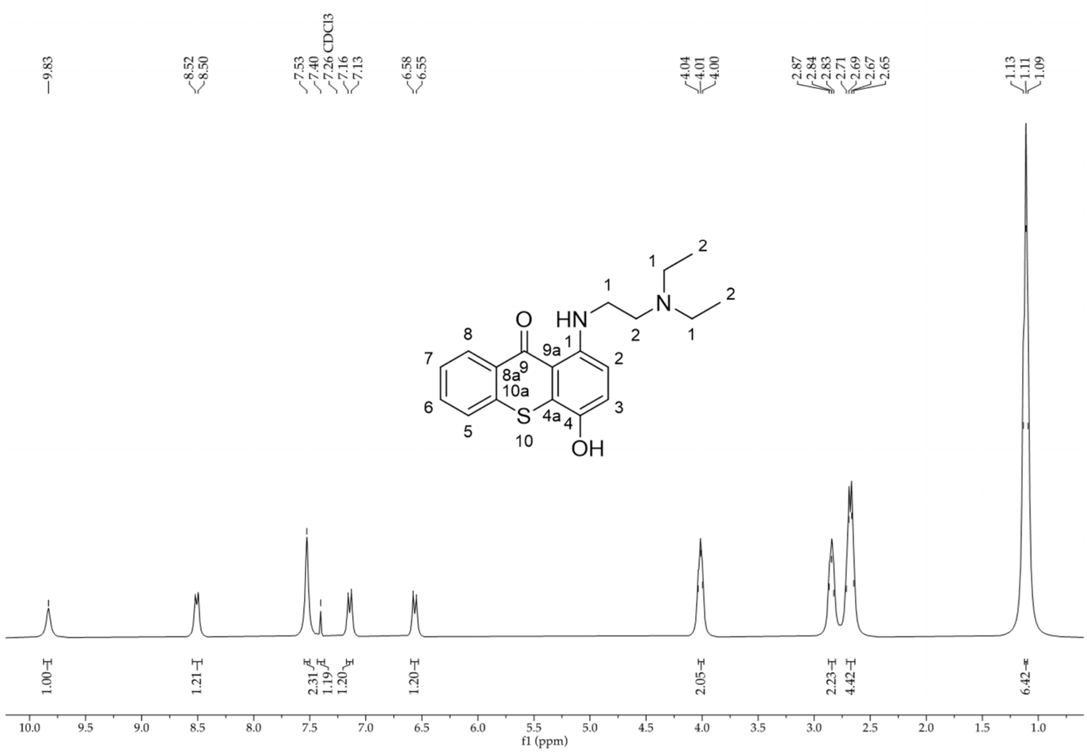

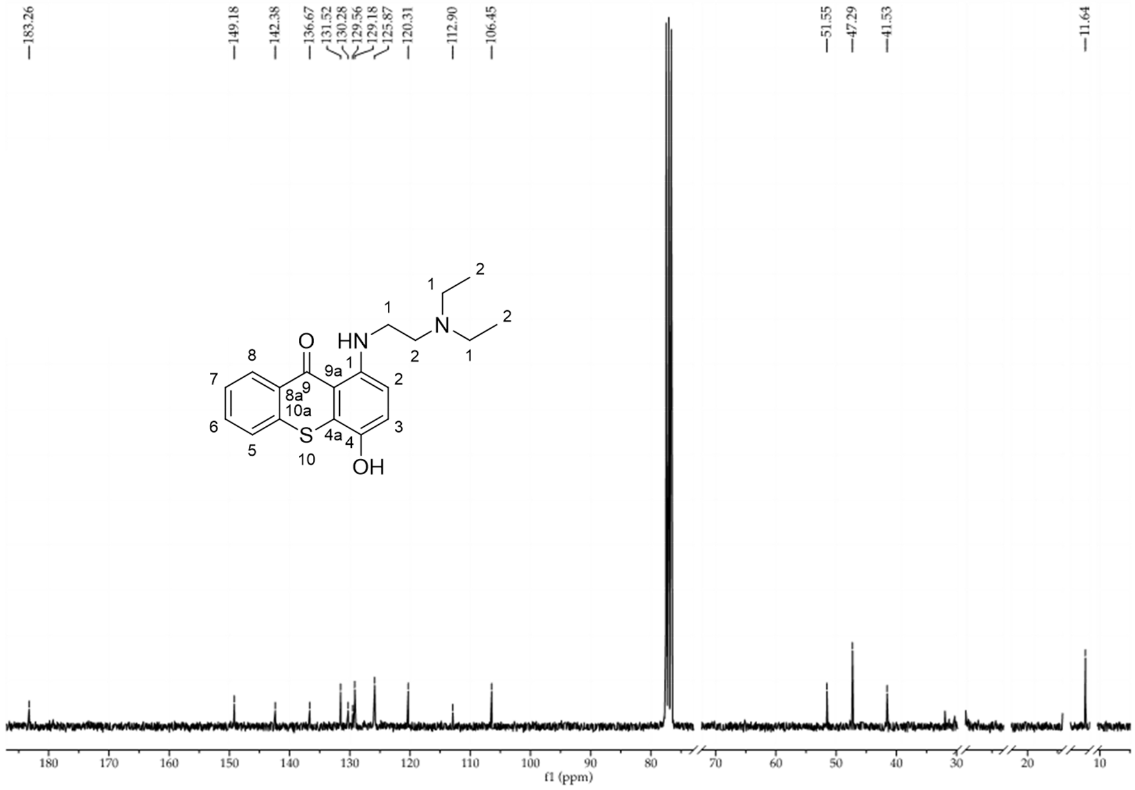

Figure S.1.1. ^{1}H NMR (top, 300.13 MHz, $CDCl_3$) and ^{13}C NMR (bottom, 75.48 MHz, $CDCl_3$) spectra of 1-{[2-(diethylamino)ethyl]amino}-4-hydroxy-9*H*-thioxanthen-9-one (**8**).

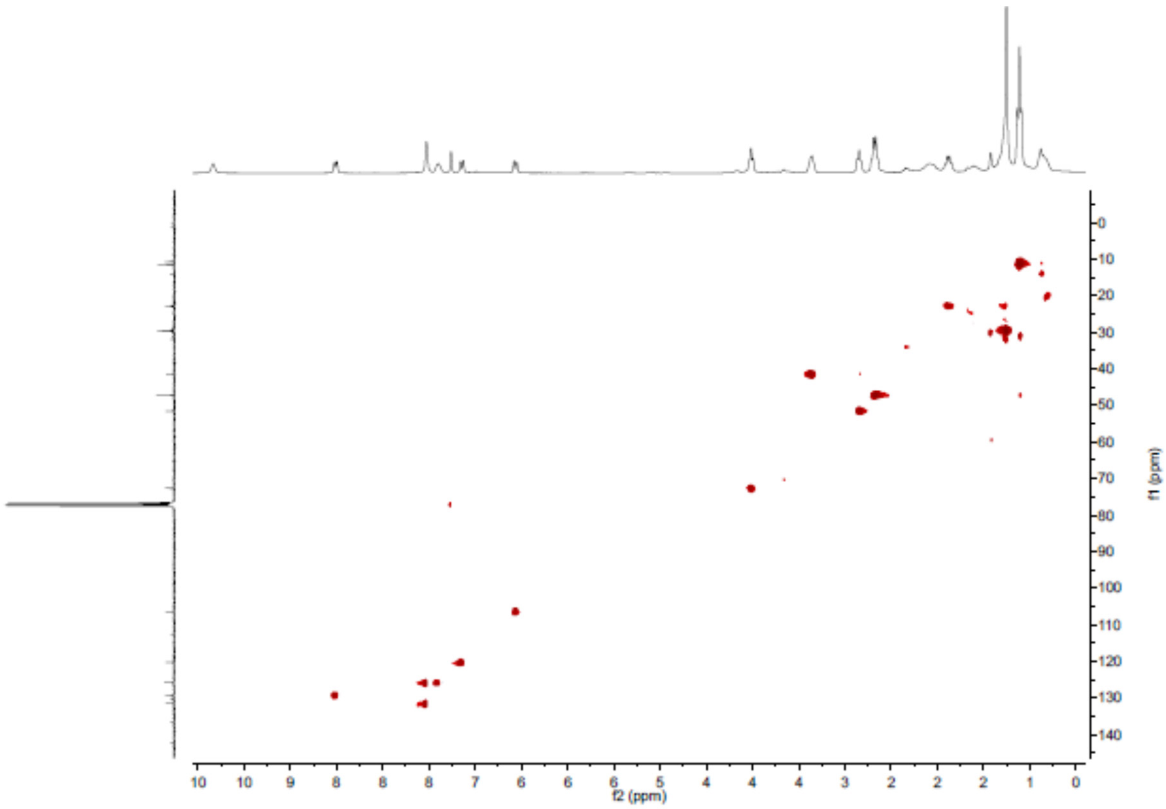

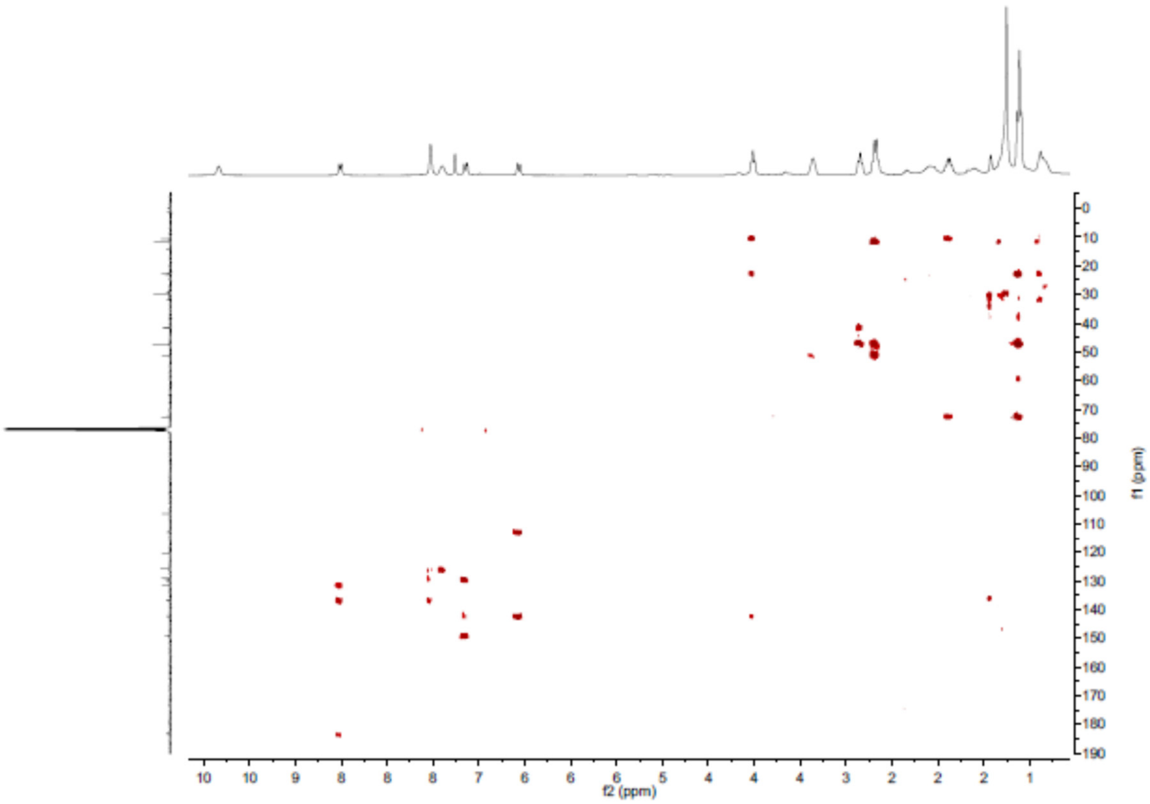

Figure S.1.2. HSQC and HMBC spectra of 1-{[2-(diethylamino)ethyl]amino}-4-hydroxy-9*H*-thioxanthen-9-one (**8**).

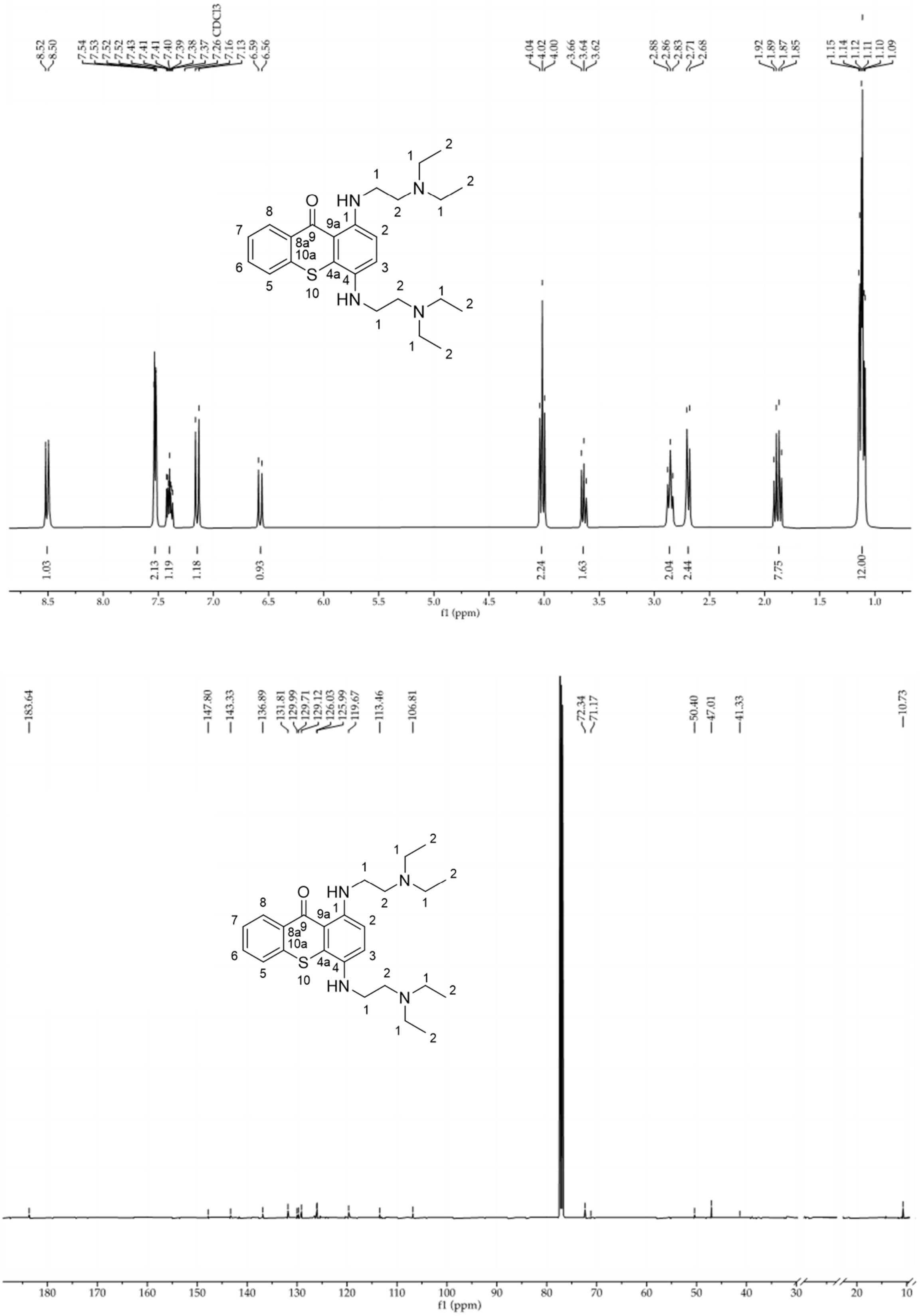

Figure S.1.3. ^{1}H NMR (top, 300.13 MHz, $CDCl_3$) and ^{13}C NMR (bottom, 75.48 MHz, $CDCl_3$) spectra of 1,4-bis{[2-(diethylamino)ethyl]amino}-9*H*-thioxanthen-9-one (**9**).

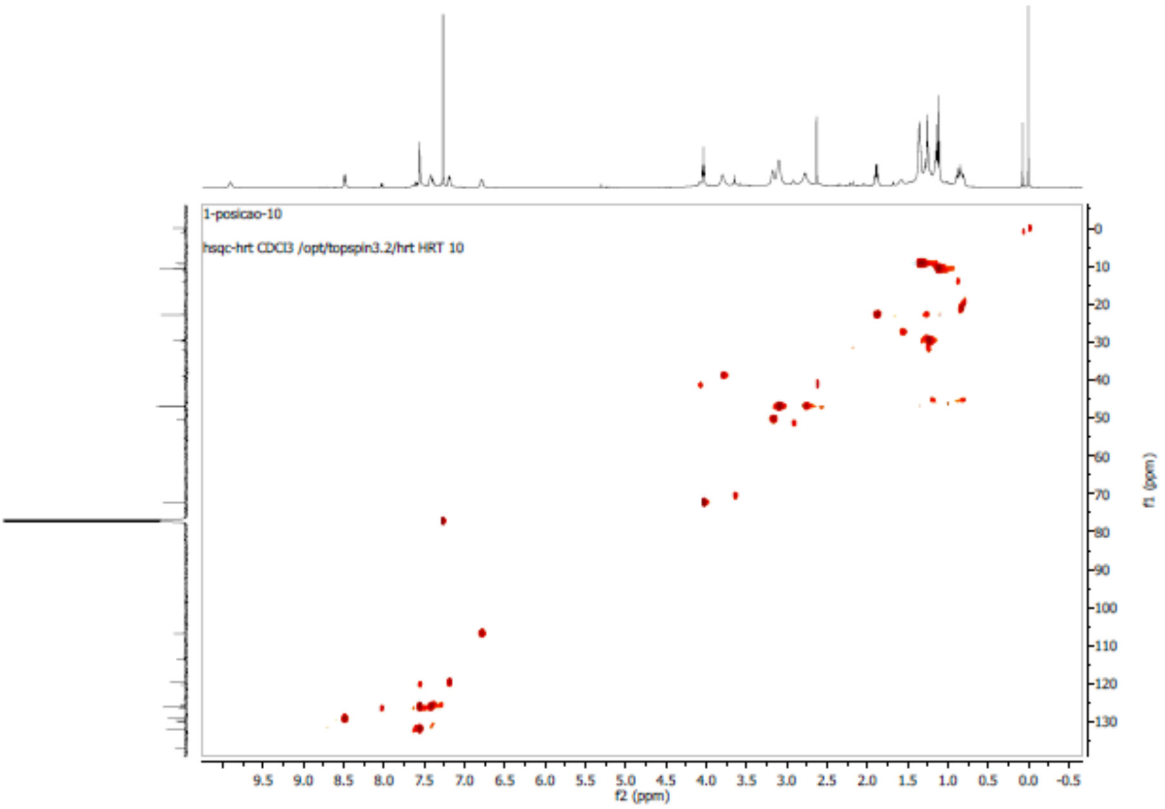

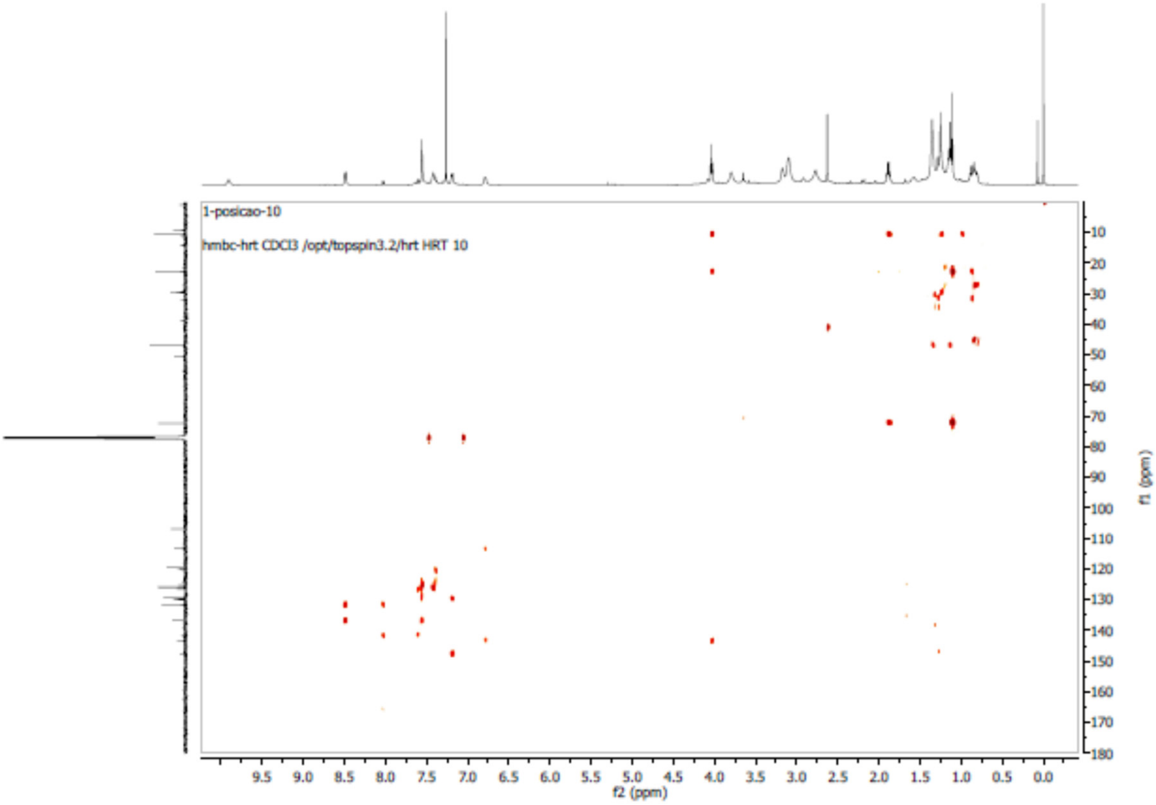

Figure S.1.4. HSQC and HMBC spectra of 1-{[2-(diethylamino)ethyl]amino}-4-hydroxy-9*H*-thioxanthen-9-one (**9**).

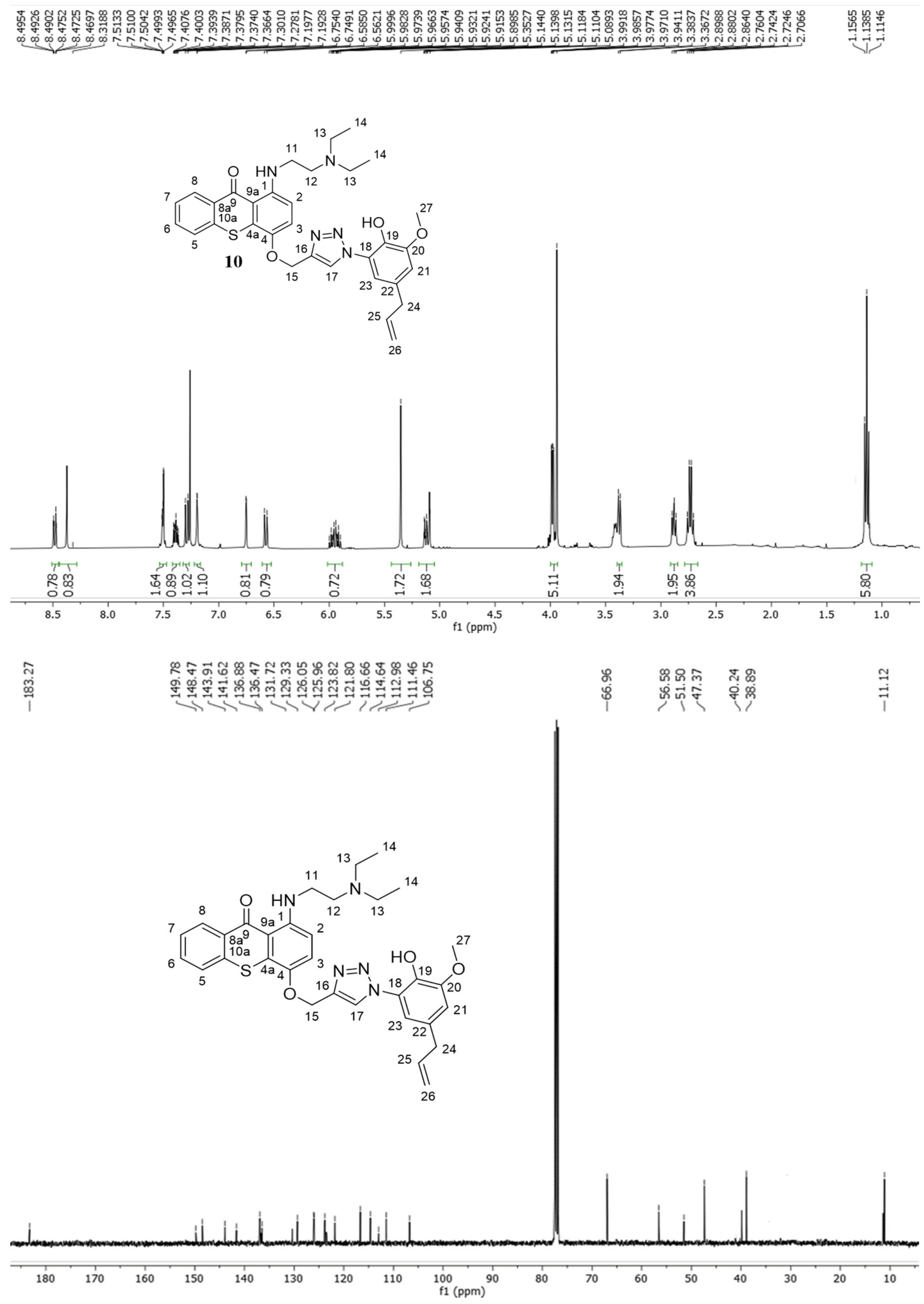

Figure S.1.5. ^{1}H NMR (top, 400 MHz, $CDCl_3$) and ^{13}C NMR (bottom, 100 MHz, $CDCl_3$) spectra of 4-((1-(5-allyl-2-hydroxy-3-methoxyphenyl)-1*H*-1,2,3-triazol-4-yl)methoxy)-1-((2-(diethylamino)ethyl)amino)-9*H*-thioxanthen-9-one (**10**).

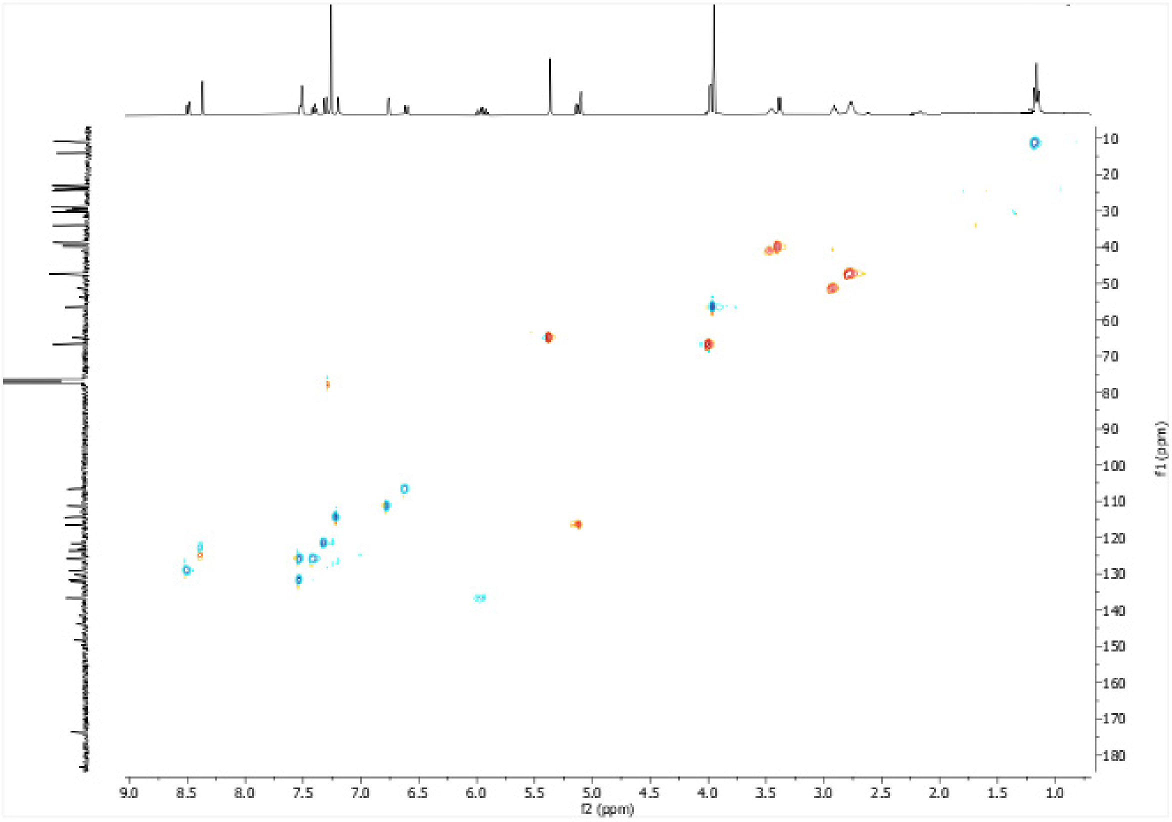

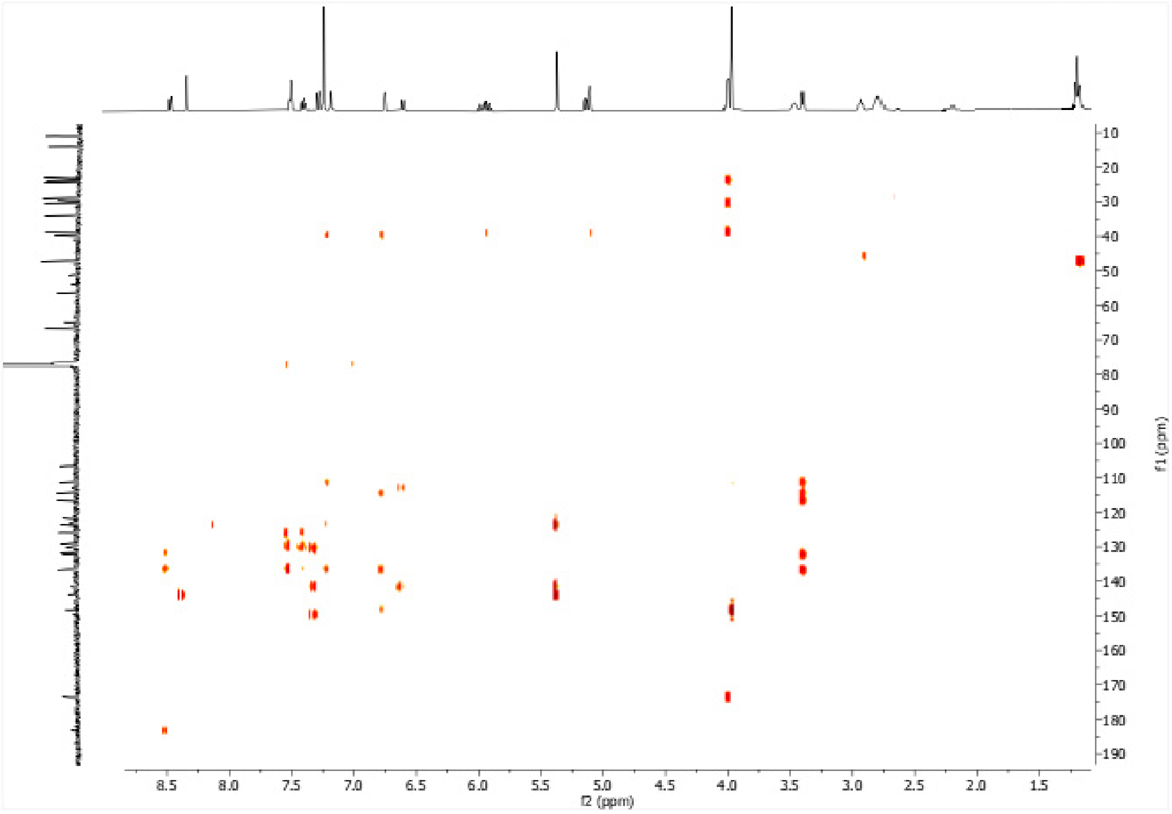

Figure S.1.6. HSQC and HMBC spectra of 1-{[2-(diethylamino)ethyl]amino}-4-hydroxy-9*H*-thioxanthen-9-one (**10**).

S.2 – HRMS spectra for compounds **8, 9**, and **10**

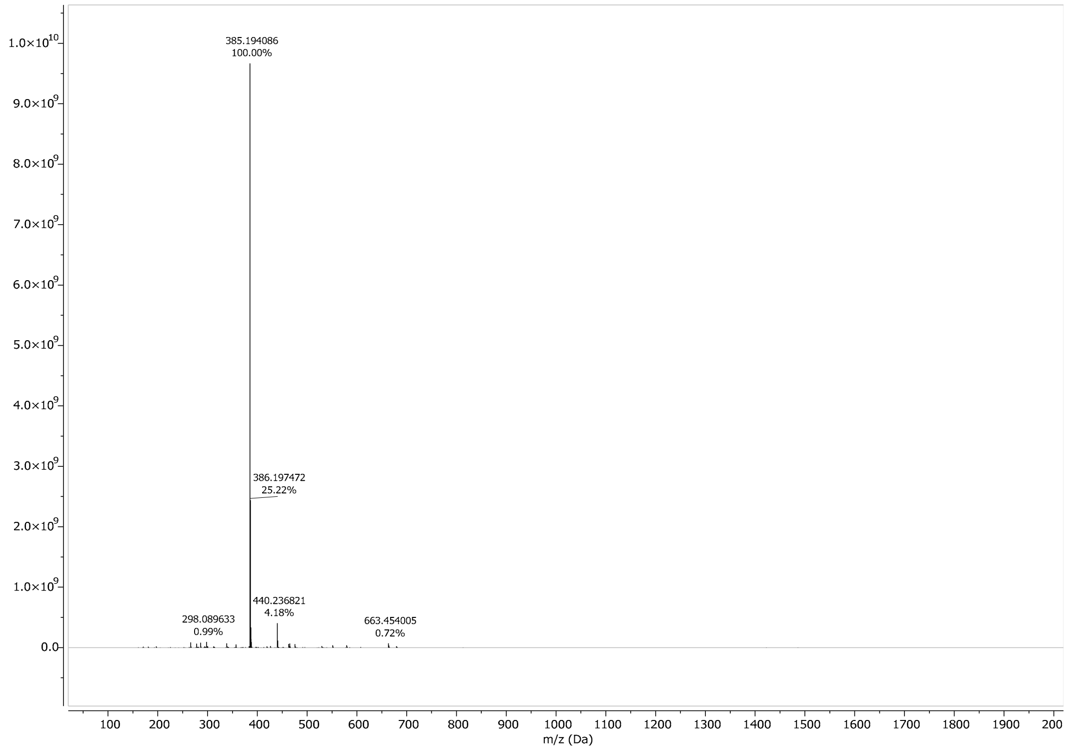

Figure S.2.1. HRMS spectrum of 1-{[2-(diethylamino)ethyl]amino}-4-hydroxy-9*H*-thioxanthen-9-one (**8**).

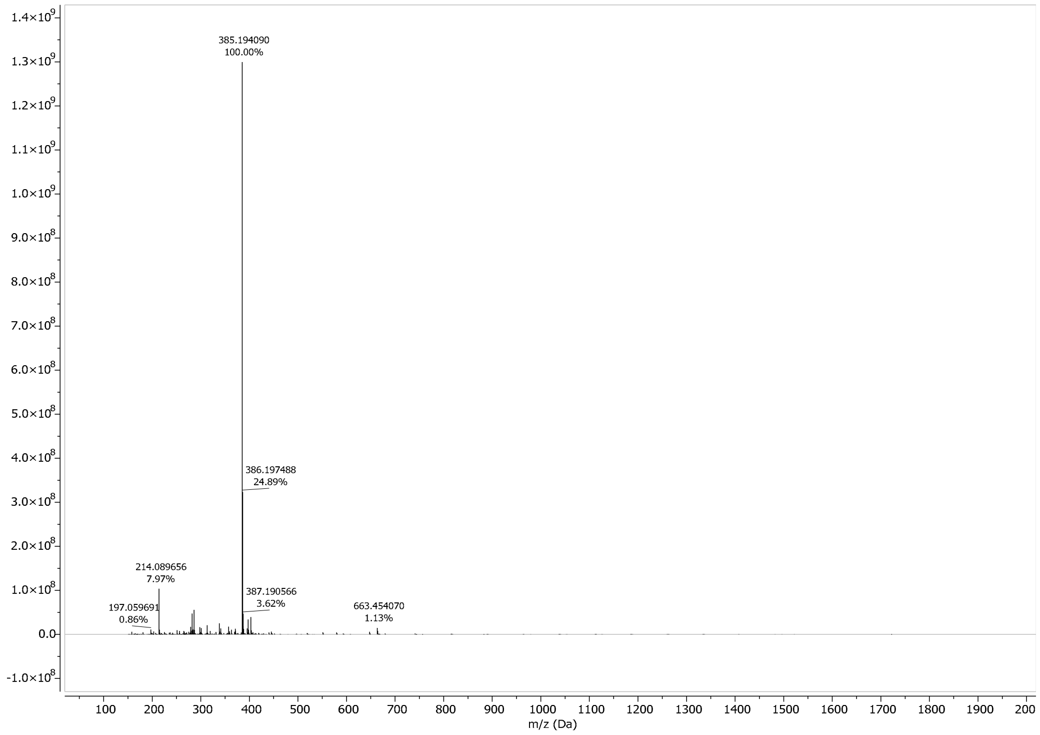

Figure S.2.2. HRMS spectrum of 1-{[2-(diethylamino)ethyl]amino}-4-hydroxy-9*H*-thioxanthen-9-one (**9**).

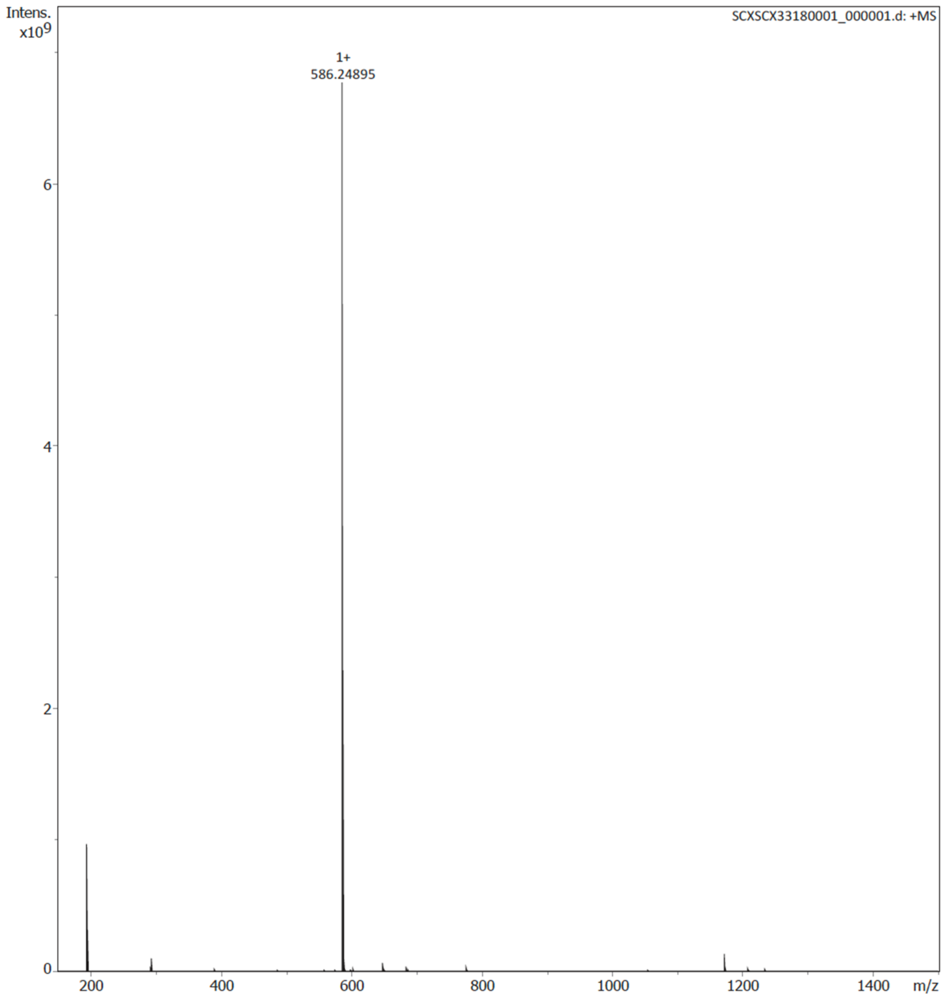

Figure S.2.3. HRMS spectrum of 4-((1-(5-allyl-2-hydroxy-3-methoxyphenyl)-1*H*-1,2,3-triazol-4-yl)methoxy)-1-((2-(diethylamino)ethyl)amino)-9*H*-thioxanthen-9-one (**10**).

S.3 - X-ray analysis of intermediate **16**

A single crystal of intermediate 4-((1-(5-allyl-2-hydroxy-3-methoxyphenyl)-1H-1,2,3-triazol-4-yl)methoxy)-1-chloro-9H-thioxanthen-9-one (**16**) was mounted on a cryoloop using paratone. X-ray diffraction data was collected at 291 K with a Gemini PX Ultra equipped with CuK_α radiation (λ = 1.54184 Å). The crystal was triclinic, space group P-1, cell volume 1212.38(17) Å^3 and unit cell dimensions a = 9.7252(8) Å, b = 9.7791(8) Å and c = 13.8940(12) Å and angles α = 69.986(7) °, β = 85.379(7)° and γ = 77.559(7) ° (uncertainties in parentheses). There are two molecules in the asymmetric unit, one molecule of **1** and one molecule of methanol, and the calculated crystal density is 1.463g/cm^{-3}. The structure was solved by direct methods using SHELXS-97 and refined with SHELXL-97 [1]. Non-hydrogen atoms of the title compound were refined anisotropically. Hydrogen atoms were either placed at their idealized positions using appropriate HFIX instructions in SHELXL and included in subsequent refinement cycles or were directly found from difference Fourier maps and were refined freely with isotropic displacement parameters. Atoms of the solvent molecule were refined with isotropic displacement parameters. The refinement converged to R (all data) = 9.21% and wR2 (all data) = 21.05%.

Full details of the data collection and refinement and tables of atomic coordinates, bond lengths and angles, and torsion angles have been deposited with the Cambridge Crystallographic Data Centre (CCDC 2191048).

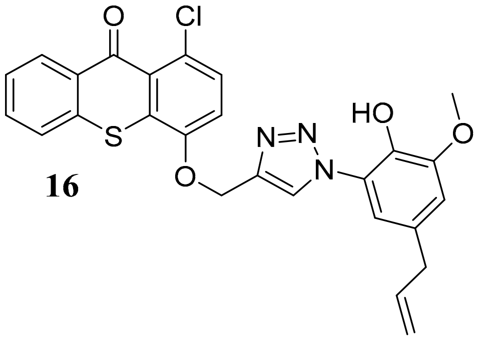

16

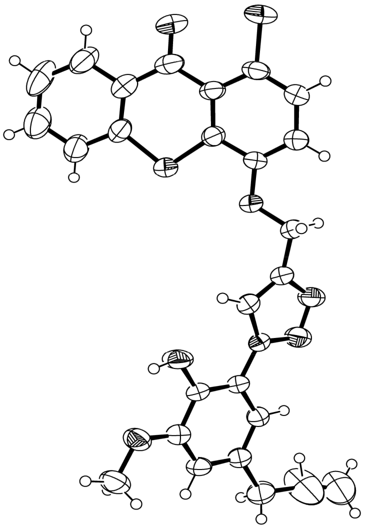

Figure S.3. The structure of 4-((1-(5-allyl-2-hydroxy-3-methoxyphenyl)-1*H*-1,2,3-triazol-4-yl)methoxy)-1-chloro-9*H*-thioxanthen-9-one (**16**) with the Ortep view.

S.4 - Docking results for compound **1** and reference compounds into the enzyme-binding site of lanosterol 14-α-demethylase.

For docking studies, the crystal structure of the enzyme lanosterol 14-α-demethylase (PDB: 5v5z) of cytochrome P450 (CYP51) was obtained from the protein database (PDB) (https://www.rcsb .org/structure/5v5z, accessed October 5). Molecular docking and, subsequent, graphical representation were performed automatically using the free software CB-Dock2 (https://cadd.labshare.cn/cb-dock2/php/index.php, accessed October 5) with default settings. Among the five pockets detected for each molecule, the lowest score value was associated with the most favourable binding conformation. Reference compounds described as inhibitors of the ergosterol biosynthetic pathway (itraconazole, ITRA and fluconazole, FL) were used as positive controls, while amphotericin B (AmB) was used as a negative control.

Table S4.1. Docking results for compound **1** and reference compounds against the enzyme lanosterol 14-α-demethylase from the pathogen *Candida albicans*.

Compounds	Docking Scores
**1**	-7.8
**ITRA**[1]	-10.4
**FL**[1]	-6.8
**AmB**[2]	20.7

[1] Itraconazole (ITRA) and fluconazole (FL) were used as positive controls. [2] Amphotericin B (AmB) was used as negative control.

a) b)

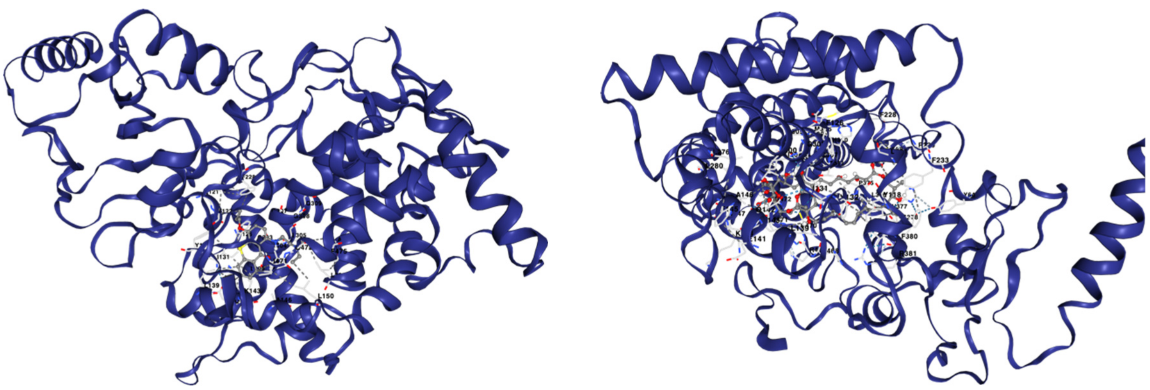

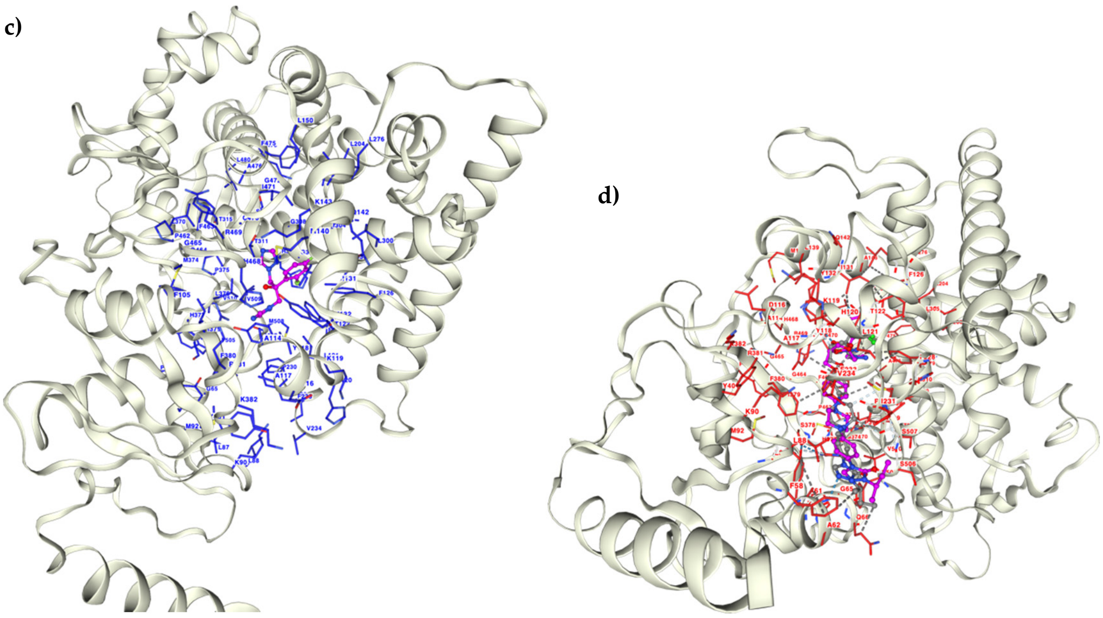

Figure S.4.2. Molecular interactions of (**a**) compound **1**, (**b**) itraconazole, (**c**) fluconazole, and (**d**) amphotericin B along the active site of lanosterol 14-α-demethylase from the pathogen *Candida albicans* (taken from CB-Dock2).
